# Antioxidants Maintain E-Cadherin Levels to Limit *Drosophila* Prohemocyte Differentiation

**DOI:** 10.1371/journal.pone.0107768

**Published:** 2014-09-16

**Authors:** Hongjuan Gao, Xiaorong Wu, LaTonya Simon, Nancy Fossett

**Affiliations:** 1 Center for Vascular and Inflammatory Diseases and the Department of Pathology, University of Maryland School of Medicine, Baltimore, MD, United States of America; 2 Department of Chemical and Biochemical Engineering, University of Maryland Baltimore County, Baltimore, MD, United States of America; University of Dayton, United States of America

## Abstract

Mitochondrial reactive oxygen species (ROS) regulate a variety of biological processes by networking with signal transduction pathways to maintain homeostasis and support adaptation to stress. In this capacity, ROS have been shown to promote the differentiation of progenitor cells, including mammalian embryonic and hematopoietic stem cells and *Drosophila* hematopoietic progenitors (prohemocytes). However, many questions remain about how ROS alter the regulatory machinery to promote progenitor differentiation. Here, we provide evidence for the hypothesis that ROS reduce E-cadherin levels to promote *Drosophila* prohemocyte differentiation. Specifically, we show that knockdown of the antioxidants, Superoxide dismutatase 2 and Catalase reduce E-cadherin protein levels prior to the loss of Odd-skipped-expressing prohemocytes. Additionally, over-expression of E-cadherin limits prohemocyte differentiation resulting from paraquat-induced oxidative stress. Furthermore, two established targets of ROS, Enhancer of Polycomb and FOS, control the level of E-cadherin protein expression. Finally, we show that knockdown of either Superoxide dismutatase 2 or Catalase leads to an increase in the E-cadherin repressor, Serpent. As a result, antioxidants and targets of ROS can control E-cadherin protein levels, and over-expression of E-cadherin can ameliorate the prohemocyte response to oxidative stress. Collectively, these data strongly suggest that ROS promote differentiation by reducing E-cadherin levels. In mammalian systems, ROS promote embryonic stem cell differentiation, whereas E-cadherin blocks differentiation. However, it is not known if elevated ROS reduce E-cadherin to promote embryonic stem cell differentiation. Thus, our findings may have identified an important mechanism by which ROS promote stem/progenitor cell differentiation.

## Introduction

Reactive oxygen species (ROS) are produced primarily in the mitochondria and increase in response to cellular stressors such as infection, starvation, or hypoxia. As a result, increased ROS levels alert the cell to changes in environmental conditions and the level of ROS correlates with the severity of stress. Consequently, high levels of ROS lead to loss of viability, whereas moderate increases promote cellular adaptation to stress [1]–[5]. In this capacity, ROS network with signal transduction pathways to direct the cellular responses to changing environmental conditions [2], [6]–[11]. For example, moderate increases in ROS promote the differentiation of many types of progenitor cells, including mammalian embryonic and hematopoietic stem cells and *Drosophila* hematopoietic progenitors (prohemocytes) [4], [8], [12]–[15]. In particular, the *Drosophila* hematopoietic system has facilitated the identification of causal links between wasp parasitization, ROS, and prohemocyte fate choice *in vivo* [16], [17].

*Drosophila* prohemocytes share key characteristics with mammalian hematopoietic stem cells, including quiescence, multipotency, and niche-dependence [18]–[21]. Prohemocytes give rise to plasmatocytes, crystal cells and lamellocytes, which are the three blood lineages in the fly [22], [23]. Plasmatocytes are operational macrophages that mediate phagocytosis of bacterial pathogens and apoptotic bodies. Crystal cells are named for their crystalline inclusion bodies, and are involved in wound healing. Lamellocytes are normally rare blood cells that are produced in large numbers in response to various types of stress signaling [24]–[28].

**Figure 1:**
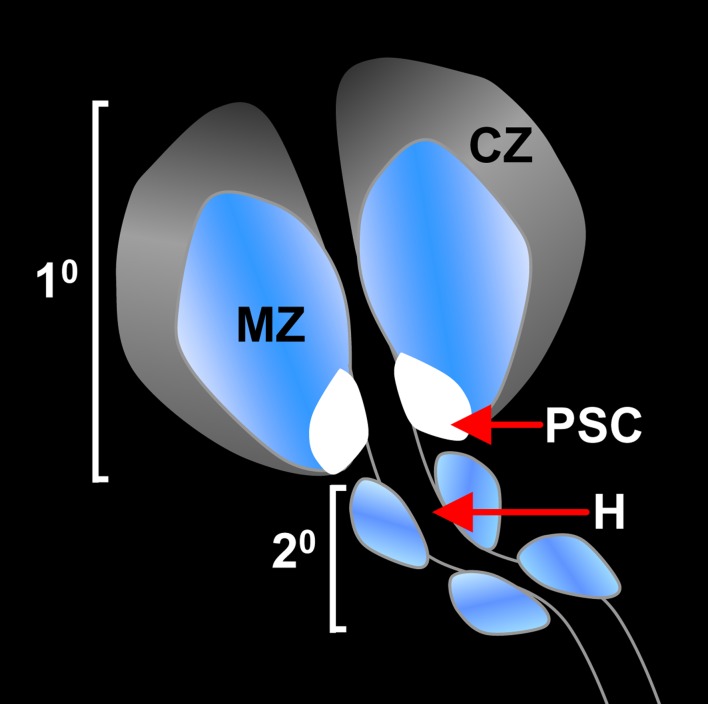
Schematic of the *Drosophila* hematopoietic lymph gland. The third larval instar lymph gland showing primary (1^0^) and secondary (2^0^) lobes. The relative positions of the three domains within the primary lobe are shown. The cortical zone (CZ) is depicted in shades of grey, the medullary zone (MZ) is depicted in shades of blue, and the stem cell niche (PSC; Posterior Signaling Center) is depicted in white. The bi-lateral lobes flank the insect heart (H). Prohemocytes reside in the MZ. Differentiating cells reside in the CZ.

Prohemocytes are located within a specialized larval organ known as the lymph gland [23], [29]. The lymph gland is specified during embryogenesis and continues to develop during the three larval instars, reaching full maturity by the mid-third larval instar [18], [19], [29], [30]. The lymph gland is a bi-lateral organ that flanks the heart and consists of one pair of primary lobes and a series of secondary lobes [22], [28]. The primary lobe is organized into three regions or zones with distinct hematopoietic functions (Figure 1). Prohemocytes reside in the medullary zone. Blood cell differentiation takes place at the periphery of the primary lobe in the cortical zone. The Posterior Signaling Center is located at the base of the primary lobe and functions as a niche to maintain prohemocyte quiescence and multipotency through the action of several signaling pathways. In addition, cortical zone hemocytes signal to the medullary zone to help maintain prohemocyte multipotency [18], [19], [22], [31]–[33]. This well-defined zonal arrangement has been instrumental in identifying the origin of various signals that regulate prohemocyte fate choice. Moreover, studies using this system have shown that increasing the level of ROS in any one of the three hematopoietic zones can drive prohemocytes to differentiate [12],[16],[17]. Nevertheless, how ROS alter the prohemocyte regulatory machinery to promote differentiation is largely unknown.

E-cadherin is the founding member of a large evolutionarily conserved family of calcium-dependent transmembrane proteins that are the principal components of adherens junctions [34], [35]. These structures are required for development and maintenance of tissue integrity [34]–[37]. We considered that ROS may reduce the level of E-cadherin and promote prohemocyte differentiation based on the following three observations: First, E-cadherin is required to maintain prohemocyte multipotency and block differentiation [38]. This function of E-cadherin is most likely conserved given that E-cadherin is also required to maintain pluripotent mammalian stem cells in an undifferentiated state [39]–[44]. Second, increased levels of ROS downregulate E-cadherin in the *Drosophila* primordial germline and in mammalian models of cancer [45]–[55]. Third, in the mature *Drosophila* ovarium, over-expression of either E-cadherin or Superoxide dismutase can prolong the lifespan of germline stem cells. However, it is not known if these factors work together to control stem cell aging [56].

In this study, we provide evidence that ROS reduce E-cadherin levels to promote *Drosophila* prohemocyte differentiation. In support of this hypothesis, we show that knockdown of Superoxide dismutatase 2 (SOD2) and Catalase (Cat) reduces E-cadherin levels. Importantly, this occurs prior to the loss of Odd-skipped- (Odd) expressing prohemocytes. Additionally, over-expression of E-cadherin limits prohemocyte differentiation resulting from paraquat-induced oxidative stress. Furthermore, FOS and the polycomb protein Enhancer of Polycomb (E(Pc)), both established targets of ROS [12], [57]–[59], control the level of E-cadherin protein expression. Finally, we show that knockdown of either SOD2 or Cat leads to increased expression of Serpent (Srp). Previously, we showed that Srp is a repressor of E-cadherin expression [38]. Thus, antioxidants and targets of ROS can control E-cadherin protein levels, and over-expression of E-cadherin can ameliorate the prohemocyte response to paraquat-induced oxidative stress. Collectively, these data suggest that elevated ROS promote prohemocyte differentiation by reducing E-cadherin protein levels. In mammalian systems, ROS promote embryonic stem cell differentiation [4], [8], [11], [15], [60], whereas E-cadherin blocks differentiation [39]–[43], [60], [61]. However, it is not known if elevated ROS reduce E-cadherin levels to promote embryonic stem cell differentiation. Thus, our findings may have identified E-cadherin as a critical component of the stem/progenitor cell response to oxidative stress.

## Materials and Methods

### Fly strains

*w^1118^* or *y w^67c23^* flies served as the wild-type stock for these studies. The following strains were generous gifts from colleagues: *UAS-E-cadherin* from G. Longmore (Washington University); *domeless-Gal4* from M. Crozatier (University Paul Sabatier); *Tep4-Gal4* from T. Tokusumi and R. A. Schulz (University of Notre Dame). The following strains were obtained from the Bloomington Stock Center: *cn^1^ shg^2^ bw^1^ sp^1^/CyO*, *y^1^ v^1^; UAS-Sod2^RNAi^*, *UAS-Sod2*; *y^1^ w^67c23^; Sod2^KG06854^*, *y^1^ v^1^; UAS-Cat^RNAi^*, *y^1^ v^1^; UAS-Jafrac^RNAi^*, *y^1^ v^1^; UAS-fos^RNAi^*, *y^1^ v^1^; UAS-E(Pc)^RNAi^*, *y^1^ v^1^; UAS-basket^RNAi^*, y^1^ sc* v^1^; UAS-ND75^RNAi^.

### Paraquat treatment

Early-third instar larvae (collected 78 to 86 hours after egg laying) were placed on media containing either 0 or 10 mM paraquat (1,1′-dimethyl-4,4′-bipyridinium dichloride; Sigma) for 6 hours. Larvae were then thoroughly washed, transferred to fresh media without paraquat, and allowed to recover from treatment for at least 18 hours prior to dissection.

### Gene expression analyses

Gene expression analyses were conducted using lymph glands from mid-third instar larvae (collected 96 to 104 hours after egg laying). However, as indicated in specific experiments, gene expression analyses were also conducted using either early-third instar larvae (collected 78 to 86 hours after egg laying) or late-third instar larvae (collected 112 to 120 hours after egg laying). All control and experimental samples were age matched and cultured on standard media at 23°C. The UAS/Gal4 binary system [62] was used to express transgenes in a tissue-specific manner. Controls for these experiments included the Gal4 drivers crossed to *w^1118^* or *y w^67c23^* mates. In general, the *dome-Gal4* driver was used for all transgene expression studies. However, the *Tep-Gal4* driver was used in experiments involving paraquat treatment because *dome-Gal4/+* and *dome-Gal4/+; UAS-E-cadherin/+* animals died after treatment.

### Immunofluorescence

The dissection and fixation of larval lymph glands were performed as previously described [63]. ROS levels in the lymph gland were detected using the superoxide specific dye, dihydroethdium (DHE, Invitrogen [12]). Rabbit anti-Odd was a generous gift from J. Skeath (Washington University School of Medicine, [64]) and used at a 1∶4,000 dilution. The following mouse antibodies directed against specific hemocyte antigens were generous gifts from I. Ando (Biological Research Center of the Hungarian Academy of Sciences) and used at the indicated dilutions: P1 (Nimrod; [65]), 1∶50 and L1 (Attilla;[66]), 1∶50. Rabbit anti-prophenoloxidase A1 (anti-ProPO) was a generous gift from F. C. Kafatos (EMBL, [67]) and used at a 1∶100 dilution. Rabbit anti-U-shaped was used at a 1∶4,000 dilution [68]. Rabbit anti-Serpent was used at a 1∶8000 dilution [68]. Rat anti-DE-cadherin was obtained from the Developmental Studies Hybridoma Bank and used at a concentration of 10 µg/ml. Alexafluor-555-, -568 or -488-conjugated secondary antibodies directed against rabbit, mouse, or rat (Invitrogen) were used at a 1∶2,000 dilution. Fluorescence was captured, analyzed, and recorded using Olympus confocal microscopy or Zeiss Axioplan optics. The relative expression of medullary zone markers was determined from the densitometric mean values calculated for fluorescent antibody staining using Zeiss Axiovision software as previously described [63]. Prohemocyte, plasmatocyte, and crystal cell counts were divided by the total primary lobe area to normalize for differences in lymph gland size. Blood cell counts were analyzed using Zeiss Axioplan software as previously described [32], [69]. The statistical significance was evaluated using the Student's t-test. In our hands, control lymph glands have an average of 1 lamellocyte per lymph gland lobe. However, lamellocytes can form large aggregates making it difficult to obtain accurate cell counts. For this reason, we scored primary lymph gland lobes positive for aberrant lamellocyte differentiation when aggregates were greater than 300 µm^2^ or more than 5 individual lamellocytes were visible [69]. Statistical significance was then evaluated using aberrant differentiation as a categorical variable for experimental and control samples in 2×2 contingency tables. P values were calculated using Fisher's Exact test. At least 20 primary lymph gland lobes were sampled, and each assay consisted of at least 10 control and 10 experimental samples.

## Results

### SOD2 is required for E-cadherin expression

Superoxide is one of the principal sources of cellular ROS, and is formed as a by-product of oxidative metabolism in the mitochondria [7]. Superoxide can undergo dismutation to form hydrogen peroxide, which is catalyzed by SOD2 [4]. In *Drosophila*, plasmatocyte differentiation increases in *Sod2/Sod2* hypomorphs during the late-third larval instar [12]. We recently showed that E-cadherin blocks plasmatocyte differentiation to maintain the prohemocyte population [38]. Finally, we showed that knockdown of the mitochondrial electron transport chain complex I protein, ND75, reduced E-cadherin protein expression. We used the *domeless-Gal4* (*dome-Gal4*) driver to express the *UAS-ND75^RNAi^* transgene in prohemocytes (Figure S1). Based on these observations, we tested if SOD2 maintains E-cadherin protein levels as a means to limit prohemocyte differentiation.

**Figure 2:**
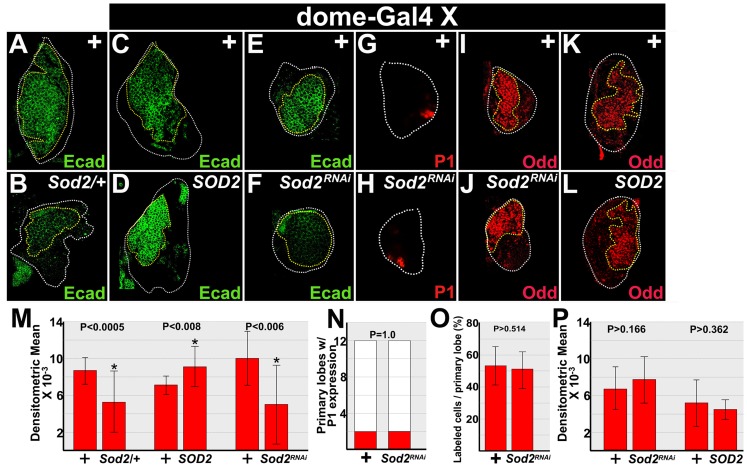
SOD2 is required to maintain E-cadherin protein expression. (**A–F**) The effect of SOD2 levels on E-cadherin protein expression. (**A–D**) Mid-third instar; (**E,F**) early-third instar. (**A,B**) E-cadherin levels were significantly reduced in the lymph glands of larvae that were heterozygous for a *Sod2* hypomorphic (*Sod2/+*) allele compared to wild-type controls (+). (**C,D**) *dome-Gal4* driven over-expression of SOD2 (*SOD2*) in prohemocytes produced a significant increase in E-cadherin expression compared to *dome-Gal4* heterozygous controls (+). (**E,F**) Additionally, *dome-Gal4* driven knockdown of SOD2 (*Sod2^RNAi^*) in prohemocytes produced a significant decrease in E-cadherin expression compared to controls. (**G,H**) *dome-Gal4* driven knockdown of SOD2 in prohemocytes did not produce a significant change in expression of the plasmatocyte marker, P1, during the early-third instar. (**I–L**) The effect of SOD2 levels on Odd protein expression. (**I,J**) Early-third instar; (**K,L**) mid-third instar. (**I,J**) *dome-Gal4* driven knockdown of SOD2 in prohemocytes did not change Odd expression levels. (**K,L**) Likewise, *dome-Gal4* driven over-expression of SOD2 in prohemocytes did not change Odd expression levels. White dotted lines delineate the entire lymph gland; yellow dotted lines delineate the prohemocyte pool (**C–L**) *dome-Gal4* females were crossed to *UAS*-*Sod2^RNAi^*, *UAS-Sod2* or wild-type (+) males. (**M**) Histogram showing the relative levels of E-cadherin in control (+) lymph glands and those with altered SOD2 expression levels; *Sod2/+* (n = 16), *SOD2* (n = 16), and *Sod2^RNAi^* (n = 18). (**N**) Histogram showing that plasmatocyte differentiation did not increase with knockdown of SOD2 in early-third larval instar lymph glands. Fisher's Exact test; P value is as shown; n = 12. (**O**) Histogram showing that the percentage of Odd-expressing cells did not decrease in lymph glands with knockdown of SOD2 (n = 18). (**P**) Histogram showing that the relative levels of Odd did not change in control (+) lymph glands and those with altered SOD2 expression levels; *Sod2^RNAi^* (n = 18), and *SOD2* (n = 14). (**M,O,P**) Student's t-test; error bars show standard deviation; P values are as shown.

Initially, we assayed the level of E-cadherin in animals that were heterozygous for a *Sod2* hypomorphic allele. Under these conditions, we observed a statistically significant reduction in E-cadherin protein levels compared to wild-type controls (Figure 2 A,B,M). We then used the UAS/Gal4 system to determine if changing SOD2 levels in medullary zone prohemocytes altered E-cadherin protein expression. Specifically, we again used *dome-Gal4* to express *UAS-SOD2* and *UAS-Sod2^RNAi^* transgenes in prohemocytes (Figure 2 C–F). Over-expression of SOD2 produced a statistically significant increase in E-cadherin protein levels compared to *dome-Gal4* heterozygous controls (Figure 2 C,D,M). Additionally, we knocked down SOD2 in early-third instar larval prohemocytes and observed that E-cadherin expression was significantly reduced compared to controls (Figure 2 E,F,M). We confirmed these results using another *UAS-Sod2^RNAi^* transgene (Figure S2). Thus, SOD2 is required to maintain E-cadherin levels in prohemocytes, and over-expression of SOD2 increases E-cadherin levels.

Having established that E-cadherin is reduced in early-third instar SOD2 knockdowns, we then tested if the reduction resulted from prohemocyte loss due to increased differentiation. First, we tested if plasmatocyte differentiation increased in the lymph glands of early-third instar SOD2 knockdowns. Under these conditions, we did not see an increase in plasmatocyte differentiation (Figure 2 G,H,N). Next, we tested for prohemocyte loss. To monitor prohemocytes, we used the specific marker, Odd because previous studies indicated that E-cadherin and Odd act in different prohemocyte regulatory pathways [38]. Therefore, Odd expression is not regulated by E-cadherin; and, in this context, changes in Odd expression result from changes in prohemocyte numbers in E-cadherin mutants. Importantly, we did not observe a reduction in the number of Odd-expressing prohemocytes between SOD2 knockdowns and controls during the early-third instar (Figure 2 I,J,O). Thus, knockdown of SOD2 leads to a reduction in E-cadherin levels prior to the loss of Odd-expressing prohemocytes. This suggests that the decrease in E-cadherin levels in early-third instar SOD2 knockdowns was not likely due to an overall reduction in the prohemocyte population. However, loss of SOD2 function did produce a reduction in the number of Odd-expressing prohemocytes during the late-third instar (Figure S3). This may have been due to the onset of prohemocyte differentiation, resulting from downregulation of E-cadherin. In support of this hypothesis, our previous work showed that knockdown of E-cadherin leads to loss of Odd-expressing prohemocytes in mid- to late-third instar lymph glands [38].

Additionally, in contrast to E-cadherin, Odd expression was not affected by altering SOD2 levels in early- and mid-third instar prohemocytes. In this regard, there was no decrease in the level of Odd expression when SOD2 was knocked down in early-third instar prohemocytes. Furthermore, over-expression of SOD2 in mid-third instar prohemocytes did not produce increased levels of Odd expression (Figure 2 I–L,P). Thus, SOD2 is required to maintain E-cadherin protein levels; however, it is not required to maintain either the number of Odd-expressing prohemocytes or level of Odd expression.

**Figure 3:**
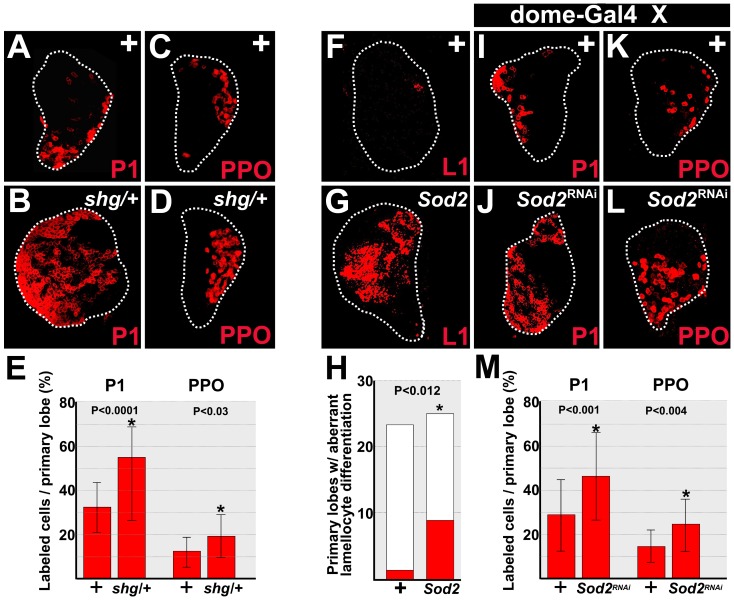
SOD2 and E-cadherin limit blood cell differentiation. (**A–E**) Reduction in the level of E-cadherin increased blood cell differentiation. (**A,B**) Plasmatocyte numbers were significantly greater in the lymph glands of animals that carry only one copy of the gene that encodes E-cadherin (*shotgun*; *shg*) compared to wild-type controls (+). (**C,D**) Likewise, crystal cell numbers significantly increased in *shg/+* lymph glands compared to controls. (**E**) Histogram showing that the percentage of plasmatocytes (P1; n = 15) or crystal cells (PPO; n = 16) was significantly greater in *shg/+* lymph glands than in controls (+). (**F–H**) Loss of SOD2 expression results in aberrant lamellocyte differentiation. (**F,G**) Lamellocyte (lm) differentiation was significantly increased in *Sod2/Sod2* hypomorphs (*Sod2*). Lamellocytes were identified using the cell-specific marker, L1. (**H**) Histogram showing that the number of primary lymph gland lobes with aberrant lamellocyte differentiation was significantly greater in *Sod2* than in controls (+). Fisher's Exact test; P value is as shown; controls, n = 24; *Sod2*, n = 26. (**I–M**) Knockdown of SOD2 increased (**I,J**) plasmatocyte and (**K,L**) crystal cell differentiation. *dome-Gal4* females were crossed to (**I,K**) control (+) or (**J,L**) *UAS-Sod2^RNAi^* males. (**M**) Histogram showing that the percentage of plasmatocytes (P1; n = 20) or crystal cells (PPO; n = 19) was significantly greater in SOD2 knockdowns compared to controls (+). Plasmatocytes were identified using the cell-specific marker, P1. Crystal cells were identified using the cell-specific marker Prophenoloxydase (PPO). White dotted lines delineate the entire lymph gland. Student's t-test; error bars show standard deviation; P values are as shown.

We previously showed that E-cadherin is required to limit the differentiation of all three blood cell types, plasmatocytes, crystal cells and lamellocytes [38]. Over-expression of E-cadherin in prohemocytes reduces the number of plasmatocytes and crystal cells. However, while knockdown of E-cadherin results in aberrant lamellocyte differentiation, it does not produce an increase in the number of plasmatocytes or crystal cells [38]. We tested if plasmatocyte and crystal cell differentiation increased in animals with only one copy of the gene that encodes E-cadherin, *shotgun* (*shg*), and we observed a statistically significant increase in both plasmatocytes and crystal cells under these conditions (Figure 3 A–E). These results confirm that E-cadherin limits plasmatocyte and crystal cell differentiation. Given that SOD2 maintains E-cadherin protein levels in prohemocytes, then SOD2 should also limit the differentiation of all three blood cell types. We examined blood cell differentiation in late-third larval instar SOD2 mutants and observed that, in addition to limiting plasmatocyte differentiation [12], SOD2 is also required to limit lamellocyte and crystal cell differentiation (Figure 3 F–M). Thus, both E-cadherin and SOD2 limit the differentiation of all three blood cell types, which is expected given that SOD2 is required to maintain E-cadherin protein levels.

### Over-expression of E-cadherin limits ROS-induced prohemocyte loss and aberrant differentiation

Our results suggest that increased levels of superoxide promote prohemocyte differentiation by reducing E-cadherin levels. If this is the case, then over-expression of E-cadherin should limit superoxide-induced differentiation. Paraquat has been widely used to increase superoxide production *in vivo* [70]. We confirmed that paraquat treatment both increased ROS levels and decreased E-cadherin expression in the lymph gland (Figure S4). The *Tep-Gal4* driver was used to over-express *UAS-E-cadherin* in prohemocytes (Tep>E-cadherin) and *Tep-Gal4* heterozygotes (Tep/+) served as controls. We identified prohemocytes using the specific marker, Odd, and assessed aberrant prohemocyte differentiation by assaying for lamellocytes.

**Figure 4:**
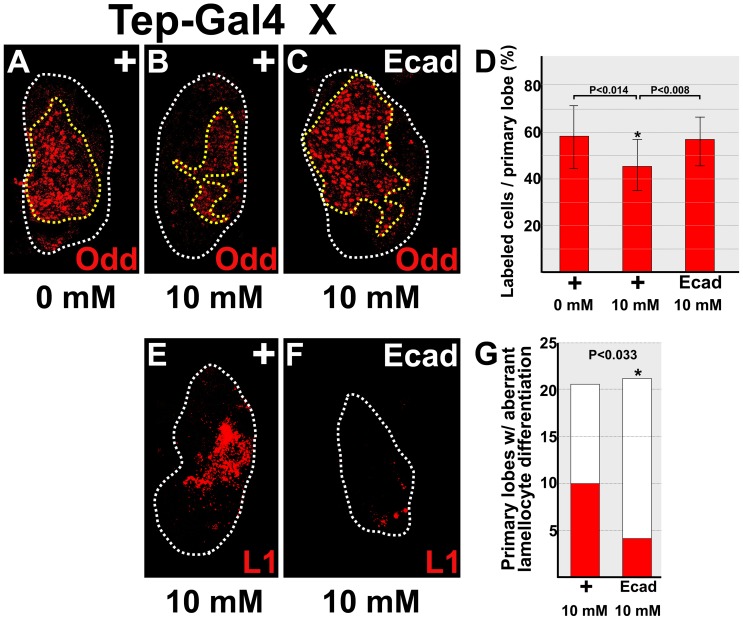
E-cadherin blocks paraquat-induced prohemocyte differentiation. (**A–D**) Over-expression of E-cadherin blocks paraquat-induced prohemocyte loss. (**A,B**) The percentage of Odd-expressing prohemocytes was significantly reduced in lymph glands of paraquat-treated (10 mM) *Tep-Gal4* heterozygotes (+) compared to untreated (0 mM) *Tep-Gal4* heterozygotes (+). (**C**) The percentage of Odd-expressing prohemocytes was significantly increased in paraquat-treated animals with *Tep-Gal4* driving *UAS-E-cadherin* (Ecad) compared to (**B**) treated *Tep-Gal4* heterozygotes. White dotted lines delineate the entire lymph gland; yellow dotted lines delineate the prohemocyte pool. (**D**) Histogram showing the percentage of Odd-expressing prohemocytes was significantly greater in untreated *Tep-Gal4* heterozygotes (+) compared to treated *Tep-Gal4* heterozygotes (+). In addition, the percentage of Odd-expressing prohemocyte was significantly greater in treated animals with *Tep-Gal4* driving *UAS-E-cadherin* (Ecad) compared to treated *Tep-Gal4* heterozygotes (+). Student's t-test; error bars show standard deviation; P values are as shown; n = 14. (**E–G**) E-cadherin limits paraquat-induced lamellocyte differentiation. (**E,F**) The number of lymph gland lobes showing aberrant lamellocyte differentiation was significantly greater in treated *Tep-Gal4* heterozygotes (+) compared to treated animals with *Tep-Gal4* driving *UAS-E-cadherin* (Ecad). White dotted lines delineate the entire lymph gland. (**G**) Histogram showing that the number of primary lymph gland lobes with aberrant lamellocyte differentiation was significantly greater in *Tep-Gal4* heterozygotes (+) compared to animals with *Tep-Gal4* driving *UAS-E-cadherin* (Ecad). Fisher's Exact test; P value is as shown; +, n = 21; Ecad, n = 23.

First, we showed that paraquat treatment reduced prohemocyte number by comparing the results from Tep/+ treated and untreated animals. Under these circumstances, we observed a statistically significant decrease in the number of prohemocytes in treated animals compared to untreated controls (Figure 4 A,B,D). Next we determined if over-expression of E-cadherin limited paraquat-induced prohemoctye loss. This was done by comparing prohemocyte numbers from paraquat-treated Tep>E-cadherin and Tep/+ larvae. In this case, we observed that the number of prohemocytes in Tep>E-cadherin larvae was significantly greater than in Tep/+ larvae (Figure 4 B,C,D). Additionally, over-expression of E-cadherin may have completely blocked prohemocyte loss given that there was no difference in prohemocyte numbers between treated Tep>E-cadherin and untreated Tep/+ animals (Figure 4 A,C,D). We also showed that over-expression of E-cadherin limited paraquat-induced lamellocyte differentiation. Lamellocytes are rarely observed in wild-type or Tep/+ lymph glands [38]. However, paraquat treatment increased lamellocyte differentiation in Tep/+ lymph glands; whereas, over-expression of E-cadherin significantly reduced lamellocyte differentiation (Figure 4 E–G). Thus, increasing the level of E-cadherin blocks loss of prohemocytes and limits aberrant differentiation in paraquat-treated animals. Collectively, these findings provide strong support for the hypothesis that ROS downregulate E-cadherin to promote prohemocyte differentiation.

### The hydrogen peroxide scavenger Catalase is required to maintain E-cadherin levels

Superoxide is a precursor of hydrogen peroxide [71]. In *Drosophila*, excess hydrogen peroxide can lead to aberrant blood cell differentiation [12] and reduction of E-cadherin expression in germline cells [46]. Hydrogen peroxide is degraded by a variety of antioxidants, including peroxidases and peroxidasins [71]. Jafrac is a peroxidasin that is expressed in the primordial germline, and is required to maintain E-cadherin protein expression and germ cell adhesion [46]. Gene expression profiles produced by our laboratory indicate that Jafrac is also expressed in the late-third instar lymph gland (unpublished data). Based on these observations, we tested if Jafrac was required to maintain E-cadherin in prohemocytes.

**Figure 5:**
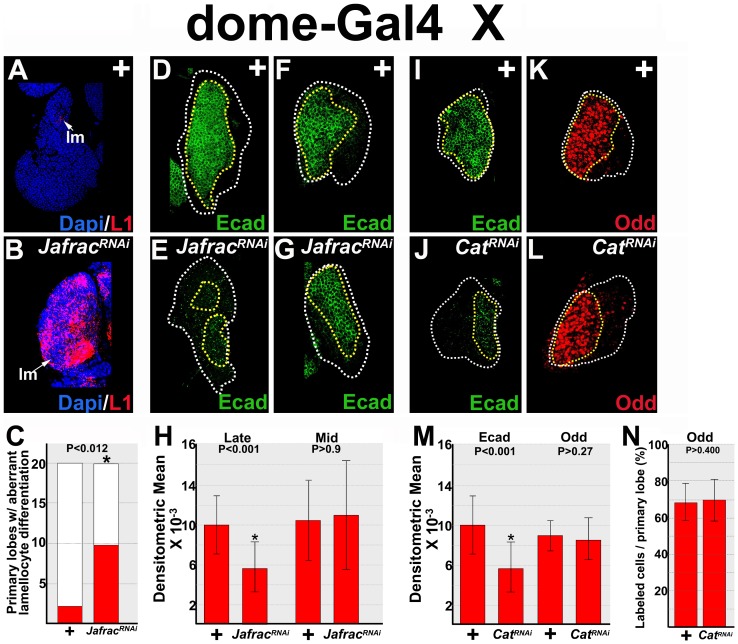
Hydrogen peroxide scavengers maintain E-cadherin protein levels. (**A–C**) Jafrac blocks lamellocyte differentiation. (**A,B**) *dome-Gal4* driven knockdown of Jafrac (*Jafrac^RNAi^*) results in aberrant lamellocyte differentiation compared to controls. (**C**) Histogram showing the number of primary lymph gland lobes with aberrant lamellocyte differentiation was significantly greater in Jafrac knockdowns than in controls. Fisher's Exact test; P value is as shown; n = 20. (**D–H**) Knockdown of Jafrac decreased levels of E-cadherin in late- but not mid-third instar lymph glands. (**D,E**) *dome-Gal4* driven knockdown of Jafrac decreased E-cadherin expression in late-third instar lymph glands compared to *dome-Gal4* heterozygous controls (+). (**F,G**) In contrast, knockdown of Jafrac did not reduce the level of E-cadherin expression during the mid-third instar. (**H**) Histogram showing the relative levels of E-cadherin in control (+) lymph glands and those with Jafrac knocked down during late- (n = 20) and mid- (n = 10) third instar. (**A–H**) *dome-Gal4* females were crossed to *UAS-Jafrac^RNAi^* (*Jafrac^RNAi^*) or wild-type (+) males. (**I–N**) Cat is required for E-cadherin, but not Odd expression in early-third instar lymph glands. *dome-Gal4* females were crossed to *UAS-Cat^RNAi^* (*Cat^RNAi^*) or wild-type (+) males. (**I,J**) *dome-Gal4* driven knockdown of Cat decreased E-cadherin levels compared to controls (+). (**K,L**) In contrast, Odd expression levels were not reduced in Cat knockdowns. White dotted lines delineate the entire lymph gland; yellow dotted lines delineate the prohemocyte pool. (**M**) Histogram showing the relative levels of E-cadherin (n = 19) and Odd (n = 15) expression in control (+) lymph glands and those with Cat knocked down during the early-third instar. (**N**) Histogram showing the percentage of Odd-expressing prohemocytes was not significantly different between control (+) and Cat knockdowns (n = 15). Student's t-test; error bars show standard deviation; P values are as shown.

We used the *dome-Gal4* driver to express the *UAS-Jafrac^RNAi^* transgene in prohemocytes. Knockdown of Jafrac led to aberrant lamellocyte differentiation and concomitant loss of E-cadherin expression in late-third instar lymph glands (Figure 5 A–E,H). However, loss of E-cadherin could have resulted from a reduction in the prohemocyte pool due to increased lamellocyte differentiation. To determine if this was the case, we knocked down Jafrac and tested if E-cadherin expression was reduced prior to the onset of aberrant lamellocyte differentiation during the mid-third instar. However, we did not observe a difference in E-cadherin protein levels between Jafrac knockdowns and controls during this developmental stage (Figure 5 F–H). Therefore, Jafrac is not required to maintain E-cadherin levels in mid-third instar prohemocytes. Consequently, we could not determine if E-cadherin levels were reduced as a direct result of increased hydrogen peroxide levels or as a by-product of prohemocyte loss due to aberrant differentiation during the late-third instar.

Given that increased hydrogen peroxide reduces E-cadherin levels in the germline, it seemed likely that this would also be the case in the lymph gland. For this reason, we tested if a different hydrogen peroxide scavenger would maintain E-cadherin levels in early-third instar prohemocytes. Cat is a conserved hydrogen peroxide scavenger [12], . In *Drosophila*, forced expression of Cat in prohemocytes blocks plasmatocyte differentiation [12]. We used *dome-Gal4* driven *UAS-Cat^RNAi^* to knockdown Cat in early-third instar prohemocytes. Under these conditions, we observed a statistically significant decrease in the level of E-cadherin (Figure 5 I,J,M). In contrast, we did not see a significant decrease in the level of Odd expression or in the number of Odd-expressing prohemocytes during this developmental stage (Figure 5 K–N). Thus, knockdown of Cat in early-third instar prohemocytes produced a decrease in the level of E-cadherin. Importantly, this occurred prior to the loss of Odd-expressing prohemocytes. Collectively, these data support the hypothesis that E-cadherin expression in the lymph gland is decreased by elevated levels of ROS and that decreased levels of E-cadherin precedes prohemocyte loss.

### Regulation of E-cadherin protein levels by ROS targets

**Figure 6:**
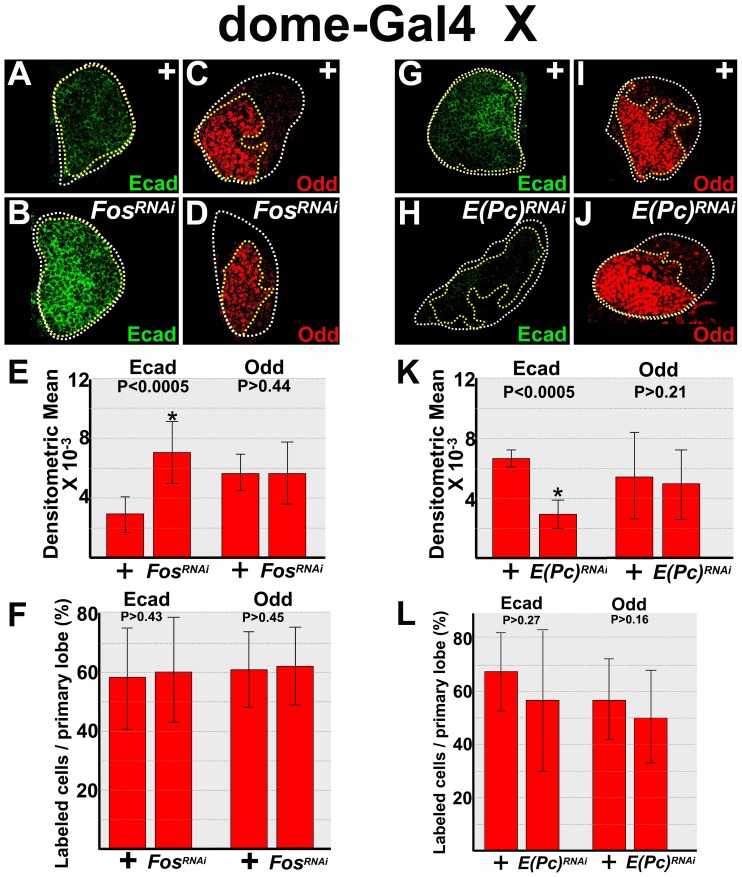
JNK targets control E-cadherin protein levels. (**A–F**) FOS represses E-cadherin, but not Odd expression in early-third instar lymph glands. *dome-Gal4* females were crossed to *UAS-kay^RNAi^* (*Fos^RNAi^*) or wild-type (+) males. (**A,B**) *dome-Gal4* driven knockdown of FOS increased E-cadherin expression compared to controls. (**C,D**) In contrast, there was no change in the level of Odd expression in FOS knockdowns. (**E**) Histogram showing the relative levels of E-cadherin (n = 15) and Odd (n = 20) expression in control (+) lymph glands and those with FOS knocked down during the early-third instar. (**F**) Histogram showing the percentage of E-cadherin- (n = 15) and Odd-expressing (n = 20) prohemocytes were not significantly different between control (+) and FOS knockdowns during the early-third larval instar. (**G–L**) E(Pc) is required for E-cadherin, but not Odd expression in early-third instar lymph glands. *dome-Gal4* females were crossed to *UAS-E(Pc)^RNAi^* (*E(Pc)^RNAi^*) or wild-type (+) males. (**G,H**) *dome-Gal4* driven *UAS-E(Pc)^RNAi^* decreased E-cadherin expression compared to controls (+). (**I,J**) In contrast, Odd expression levels were not reduced in E(Pc) knockdowns. White dotted lines delineate the entire lymph gland; yellow dotted lines delineate the prohemocyte pool. (**K**) Histogram showing the relative levels of E-cadherin (n = 19) and Odd (n = 15) expression in control (+) lymph glands and those with E(Pc) knocked down during the early-third instar. (**L**) Histogram showing the percentage of E-cadherin- (n = 19) and Odd-expressing (n = 15) prohemocytes was not significantly different between control (+) and E(Pc) knockdowns during the early-third larval instar. Student's t-test; error bars show standard deviation; P values are as shown. White dotted lines delineate the entire lymph gland; yellow dotted lines delineate the prohemocyte pool.

The Jun N-terminal Kinase (JNK) signal transduction pathway is activated by a variety of environmental stress signals, including elevated levels of ROS [12], [58], [59], [72]. Downstream effectors of this pathway include FOS/Jun heterodimers, which are a subset of the Activating protein-1 (AP-1) family of conserved transcriptional regulators [12], [58], [72], [73]. FOS is required for lamellocyte differentiation [74]; whereas E-cadherin blocks lamellocyte differentiation in response to paraquat-induce oxidative stress increased superoxide levels (Figure 4 E–G). This result prompted us to ask if FOS represses E-cadherin levels in the lymph gland and whether loss of FOS leads to increased levels of E-cadherin. To test this hypothesis, we knocked down FOS in early-third instar prohemocytes. We showed that this resulted in a statistically significant increase in the level of E-cadherin (Figure 6 A,B,E). Thus, FOS represses E-cadherin levels in prohemocytes. However, there was no increase in Odd expression under these conditions, nor was there an increase in the number of Odd- or E-cadherin expressing prohemocytes (Figure 6 C–F). This strongly suggests that increased E-cadherin levels in FOS knockdowns were not due to an increase in prohemocyte number.

JNK signaling also downregulates polycomb activity [75]. Similar to E-cadherin, loss of the polycomb protein E(Pc) leads to an increase in lamellocyte differentiation [12], [38]. Based on these observations, we tested if E(Pc) is required for E-cadherin expression. We knocked-down E(Pc) expression in early-third instar prohemocytes. This resulted in a statistically significant reduction in the level of E-cadherin (Figure 6 G,H,K). In contrast, loss of E(Pc) had no effect on the level of Odd expression or the number of E-cadherin- or Odd-expressing prohemocytes (Figure 6 I–L). Thus, E-cadherin is reduced prior to the loss of Odd-expressing prohemocytes, which strongly suggests that reduced levels of E-cadherin were not due to a reduction in the prohemocyte pool. Overall, these data show that the JNK downstream targets, FOS and E(Pc), control E-cadherin protein levels in *Drosophila* prohemocytes. Given that increased ROS activate JNK signaling, these findings are consistent with the hypothesis that elevated ROS repress E-cadherin.

GATA factors regulate gene expression during a variety of biological processes across taxa. Furthermore, GATA activity is modified through its interaction with the transcriptional co-factor, Friend of GATA (FOG). We recently showed that the GATA factor, Srp, and the FOG factor, U-shaped (Ush), regulate E-cadherin expression in prohemocytes. Srp represses E-cadherin to promote prohemocyte differentiation. However, when Srp is bound to Ush, the capacity to repress E-cadherin is diminished. Thus, with the downregulation of Ush, the amount of unbound Srp increases, which leads to the reduction of E-cadherin [38].

**Figure 7:**
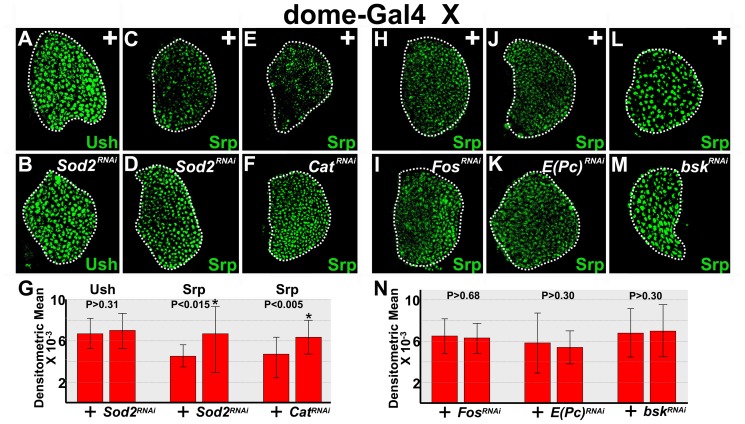
Knock down of either SOD2 or Cat increases the level of Srp expression. (**A–D**) Loss of SOD2 function increased levels of Srp but not Ush in early-third instar lymph glands. *dome-Gal4* females were crossed to *UAS*-*Sod2^RNAi^* or wild-type (+) males. (**A,B**) There was no difference in Ush expression levels in SOD2 knockdowns (*Sod2^RNAi^*) compared to controls (+). (**C,D**) In contrast, *dome-Gal4* driven knockdown of SOD2 increased Srp expression compared to controls (+). (**E,F**) Additionally, *dome-Gal4* driven knockdown of Cat (*Cat^RNAi^*) increased Srp expression compared to controls (+). *dome-Gal4* females were crossed to *UAS*-*Cat^RNAi^* or wild-type (+) males. (**G**) Histogram showing the relative levels of Ush (n = 16) and Srp (*Sod2^RNAi^*, n = 19; *Cat^RNAi^*, n = 18) expression in control (+) lymph glands and those with antioxidants knocked down during the early-third instar. Student's t-test; error bars show standard deviation; P values are as shown. (**H–N**) JNK downstream targets do not control Srp expression levels. *dome-Gal4* females were crossed to (**H,J,L**) wild-type (+) or (**I**) *UAS-Fos^RNAi^* (*Fos^RNAi^*), (**K**) *UAS-E(Pc)^RNAi^* (*E(Pc)^RNAi^*), or (**M**) *UAS-bsk^RNAi^* (*bsk^RNAi^*) males. Knockdown of (**H,I**) FOS, (**J,K**) E(Pc), or bsk (**L,M**) did not produce a significant change in the level of Srp expression. (**N**) Histogram showing the relative level of Srp expression in control (+) lymph glands and those with FOS (n = 17), E(Pc) (n = 19), or bsk (n = 18) knocked down during the early-third instar. Student's t-test; error bars show standard deviation; P values are as shown. White dotted lines delineate the entire lymph gland.

We asked if increasing the level of ROS repressed E-cadherin by either upregulating Srp or downregulating Ush. To test this hypothesis, we knocked down SOD2 in early-third larval instar prohemocytes and assessed Ush and Srp expression levels. Knockdown of SOD2 did not alter the level of Ush expression (Figure 7 A,B,G). In contrast, knockdown of SOD2 produced a statistically significant increase in the level of Srp expression (Figure 7 C,D,G). We confirmed these results using another *UAS-Sod2^RNAi^* transgene (Figure S5). Additionally, we observed that knockdown of Cat also resulted in a significant increase in the level of Srp expression (Figure 7, E–G). Collectively, these data suggest that increased levels of ROS upregulate Srp.

During *Drosophila* embryogenesis, GATA regulation and JNK signaling act independently to control development [76]. On the other hand, GATA factors influence Wnt activation of JNK signaling during vertebrate cardiomyocyte differentiation [77]. These observations, coupled with our data showing that both Srp and JNK downstream effectors control the level of E-cadherin, led us to consider if FOS and E(Pc) control Srp expression as a means to regulate E-cadherin levels. With the knockdown of either FOS or E(Pc) in prohemocytes, we did not observe a change in Srp expression levels (Figure 7 H–K,N). However, it was possible that Srp was upregulated by JNK signaling, but acts upstream or parallel to FOS and E(Pc). To determine if this was the case, we evaluated Srp expression in JNK mutants. We knocked down the JNK homologue, *basket* (*bsk*), and did not observe a significant change in the level of Srp (Figure 7 L–N). Collectively, these results suggest that Srp acts independently of JNK signaling.

## Discussion

In this report, we showed that SOD2 and Cat maintain E-cadherin protein expression in early-third instar prohemocytes, but are not required to maintain the population of Odd-expressing prohemocytes during this developmental stage. These results strongly suggest that elevated ROS lower E-cadherin levels prior to the onset of prohemocyte differentiation. Thus, reduction in the level of E-cadherin is likely one of the initial steps in the response of prohemocytes to increased ROS. Additionally, we showed that over-expression of E-cadherin restricts prohemocyte differentiation resulting from paraquat-induced oxidative stress. Given that E-cadherin maintains prohemocyte multipotency [38], these new findings suggest that reducing E-cadherin levels may be an important mechanism whereby ROS signaling promotes prohemocyte differentiation. As a result, E-cadherin may be a key component of the prohemocyte stress-response regulatory network.

We also showed that downstream effectors of JNK signaling, FOS and E(Pc), control the level of E-cadherin protein expression. Specifically, E(Pc) maintains E-cadherin, whereas FOS represses E-cadherin. Furthermore, these effects were detected prior to the onset of Odd-expressing prohemocyte loss. Our findings strongly suggest that FOS and E(Pc) control prohemocyte differentiation, at least in part, by regulating E-cadherin protein levels. As a result, our findings provide additional support for ROS-induced control of E-cadherin levels through networking with two established targets of ROS. Notably, all three factors, FOS, E(Pc), and E-cadherin, regulate the differentiation of lamellocytes. FOS is required for lamellocyte differentiation [74]; whereas, E(Pc) and E-cadherin block lamellocyte differentiation [12], [38]. Lamellocytes differentiate in response to various types of stress signaling, but are rarely observed under steady state conditions [24]–[28]. Thus, E(Pc) likely acts to maintain the level of E-cadherin to block lamellocyte differentiation. However, downregulation of E(Pc) and upregulation of FOS would reduce the level of E-cadherin to promote lamellocyte differentiation. Interestingly, we also showed that loss of either SOD2 or Cat increased levels of Srp; however, Srp is not likely regulated by JNK signaling. Our previous work showed that over-expression of Srp downregulates E-cadherin and promotes prohemocyte differentiation [38]. Thus, there may be two independent mechanisms that reduce E-cadherin to promote differentiation in response to elevated ROS.

**Figure 8:**
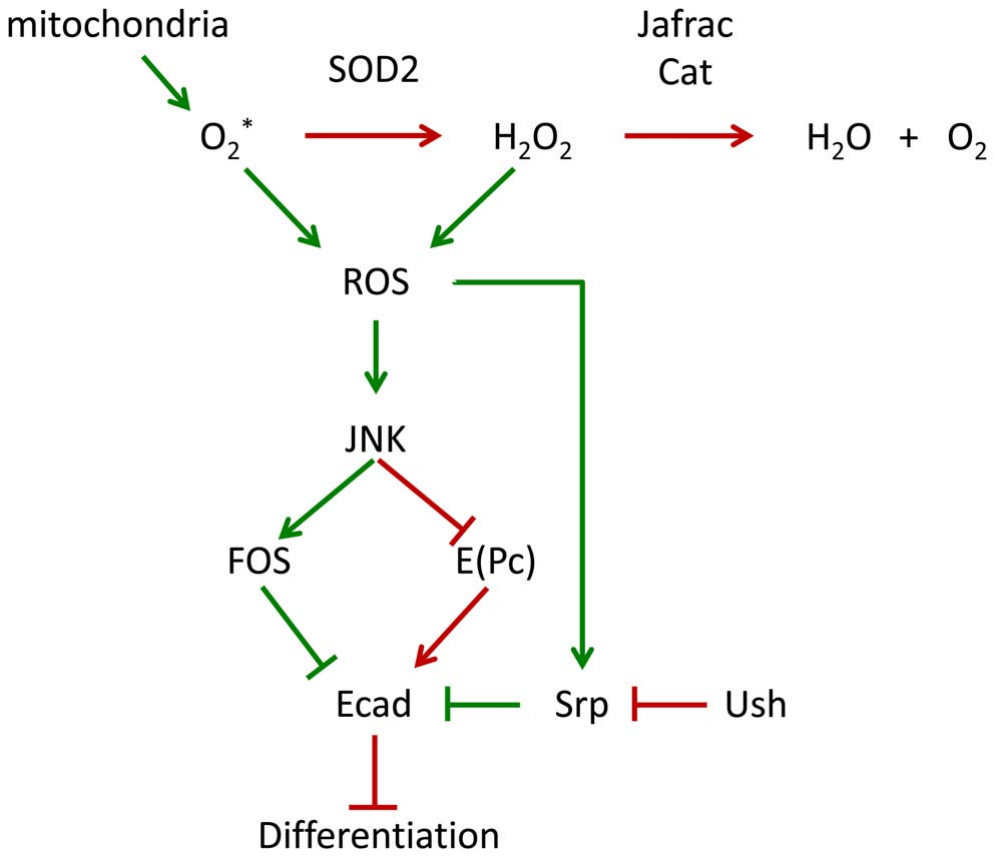
Proposed model of ROS-induced prohemocyte differentiation. Superoxide is generated in the mitochondria, and is the major source of cellular ROS. Superoxide undergoes dismutation to form hydrogen peroxide by the action of Superoxide dismutatase. Hydrogen peroxide is further degraded by the action of Jafrac and Cat to form water and oxygen. Increased levels of ROS can activate JNK signaling, which upregulates FOS and downregulates E(PC). This can reduce E-cadherin (Ecad) levels and promote prohemocyte differentiation. Upregulation of Srp by increased levels of ROS also downregulates E-cadherin to promote differentiation. Under steady state conditions, E(PC) maintains E-cadherin levels. In addition, Ush interacts with Srp to block its ability to downregulate E-cadherin. This model describes a causal link between elevated ROS, loss of E-cadherin and differentiation, which may be an important determinant of prohemocyte fate choice. Green lines mark pathways that lead to differentiation; red lines mark pathways that block differentiation.

The results presented here extend the previous model of ROS-induced prohemocyte differentiation. In this new model, increased levels of ROS upregulate both Srp and JNK signaling to reduce E-cadherin and, thereby, promote differentiation (Figure 8). While our results provide evidence that ROS reduce E-cadherin to promote prohemocyte differentiation, our model represents only part of a highly complex network that controls the hematopoietic response to ROS signaling in the fly. Recent reports have shown that wasp parasitization of larvae increases levels of ROS in the stem cell niche, which leads to increased epidermal growth factor signaling and prohemocyte differentiation [16]. Wasp parasitization also reduces Notch signaling, which increases ROS levels in differentiating blood cells [17]. Whether these signal transduction pathways interface with the Srp/E-cadherin cascade is not yet known. Finally, mitochondrial superoxide is one of the major sources of cellular ROS and is converted to hydrogen peroxide by SOD2 [7]. However, we cannot rule out the possibility that the genetic manipulation of either SOD2 or Cat alters ROS levels that originate from sources other than the mitochondria.

### Hydrogen peroxide and the downregulation of E-cadherin

We showed that Cat is required to maintain E-cadherin in early-third instar prohemocytes. In contrast, Jafrac is not required to maintain E-cadherin, either directly or indirectly, until at least after the mid-third instar. Notably, ROS levels increase in prohemocytes from the early- to late-third instar [12]. As a result, Jafrac activity may increase after the mid-third instar to maintain redox homeostasis as ROS levels rise. Thus, Cat may function to maintain homeostasis in early-third instar prohemocytes, and additional antioxidants, such as Jafrac, may be required as ROS levels increase with prohemocyte age. Interestingly, Cat is expressed in rat oligodendrocytes throughout development; whereas, Glutathione peroxidase is upregulated as these cells mature, making them more resistant to ROS [78]. Thus, a recurring regulatory strategy may involve a basal antioxidant activity that functions primarily during early development, followed by the activation of additional antioxidants as cells age.

### JNK targets and E-cadherin expression across taxa

We showed that FOS, a downstream target of JNK signaling, represses *Drosophila* E-cadherin protein expression in the lymph gland. Studies using mammalian systems have shown that FOS downregulates E-cadherin gene expression. In breast cancer cell lines, FOS has been shown to upregulate the E-cadherin transcriptional repressor, ZEB [79]. Additionally, in murine tumorigenic epithelial cell lines, FOS methylates the E-cadherin promoter and thereby blocks gene expression [80]. While it has not been established that FOS represses E-cadherin gene expression in the lymph gland, the overall capacity of FOS to antagonize E-cadherin appears to be evolutionarily conserved.

The polycomb protein E(Pc), another downstream target of JNK signaling, most likely maintains E-cadherin by silencing genes involved in E-cadherin repression. Importantly, both polycomb activity and E-cadherin are required to establish and maintain mammalian pluripotent stem cells [39]–[43], [60], [61], [81]–[83]. Thus, we may have identified a novel conserved mechanism that maintains E-cadherin to promote stem/progenitor cell potency. On the other hand, aberrant over-expression of polycomb proteins blocks E-cadherin expression to promote mammalian tumor formation [81], [82]. As a result, our findings may provide new avenues to investigate, specifically, how polycomb proteins regulate stem cell pluripotency and how dysregulation leads to cancer. This approach may also increase the utility of embryonic stem cells and induced pluripotent stem cells by reducing their inherent potential for oncogenesis [9], [61].

### ROS and the regulation of Srp transcriptional activity

There are three mammalian hematopoietic GATA factors, GATA-1, -2 and -3 [84]–[88]. GATA-2 functions to maintain the hematopoietic stem cell (HSC) population [86], [89]–[91], and all three GATA factors function later in hematopoiesis to control lineage commitment and differentiation of specific blood cell types [84], [86], [92], [93]. In *Drosophila*, Srp performs the functions of all three GATA factors in that it is required to maintain the prohemocyte pool [30], [94] and acts later to direct blood lineage commitment and differentiation [95]–[99]. We previously demonstrated that over-expression of Srp promotes lamellocyte differentiation by downregulating E-cadherin [38]. Here we showed that knockdown of either SOD2 or Cat increased Srp expression. Collectively, these findings suggest that ROS signaling upregulates Srp to promote prohemocyte differentiation. This role for Srp is strikingly similar to the one for GATA-3 in Long Term HSCs (LT-HSCs), which sustain life-long production of all mammalian blood lineages [100]. Recent studies in mice suggest that stress signaling activates GATA-3 in LT-HSCs, which interferes with self-renewal and promotes differentiation [101], [102]. Thus, in both flies and mice, stress increases GATA activity to promote hematopoietic progenitor differentiation. GATA factors also regulate a variety of biological processes across taxa. As a result, GATA activation in response to stress signaling may be a general, rather than a hematopoietic-specific response. In support of this notion, elevated ROS upregulate GATA transcription factor expression in *Caenorhabditis elegans* and in tissue culture models of cardiomyocyte differentiation [103]–[105]. Thus, one conserved GATA function may be to mediate the stress response.

GATA factors interact with FOG proteins to regulate gene expression across tissues and taxa [86]. In *Drosophila*, Srp binds to Ush to form a GATA∶FOG complex that regulates hematopoiesis [86], [98], [106]. Although knockdown of SOD2 increased Srp expression, it did not change the level of Ush expression. This suggests that increased ROS has no effect on Ush expression. However, Srp has been shown to upregulate *ush* gene expression during hematopoiesis [68], [97]. Thus, it is possible that increased ROS does in fact downregulate Ush, but the decrease is obscured by an increase in *ush* gene expression driven by an increase in Srp activity. If this is the case, then Srp upregulation of *ush* could produce a negative feedback loop that promotes GATA∶FOG complex formation and thereby prevents excessive prohemocyte differentiation. This is supported by our previous work that showed when Srp binds Ush it cannot block E-cadherin expression or promote prohemocyte differentiation [38]. Notably, studies in mice suggest that the GATA∶FOG complex is required for recovery from anemia-induced oxidative stress [107], [108]. Thus, the GATA∶FOG complex may activate regulatory pathways that promote cellular protection and recovery from oxidative stress across taxa.

In summary, we present evidence that reduction of E-cadherin is necessary to promote differentiation in response to oxidative stress. Furthermore, our studies suggest that both JNK signal transducers and GATA transcriptional activity mediate ROS-induced downregulation of E-cadherin. Given the conservation of E-cadherin function between *Drosophila* prohemocytes and mammalian pluripotent stem cells [38], our studies may have identified an important conserved mechanism by which elevated ROS promote progenitor differentiation. Importantly, this would open avenues to investigate the underlying regulatory strategies that control progenitor cell fate choice in response to stress.

## Supporting Information

Figure S1 **Knockdown of ND75 in prohemocytes reduces E-cadherin expression.** E-cadherin expression is greater in (**A**) control than in (**B**) ND75 knockdown (ND75^RNAi^) lymph glands. *dome-Gal4* females were crossed to *UAS*-*ND75^RNAi^* or wild-type (+) males. Yellow dotted lines delineate the entire lymph gland; white dotted lines delineate the prohemocyte pool. (**C**) Histogram showing the relative level of E-cadherin expression was significantly greater in control (+) lymph glands than in those with knockdown of ND75. (**D**) Histogram showing the percentage of E-cadherin-expressing prohemocytes was significantly reduced in ND75^RNAi^ lymph glands compared to controls (+). Student's t-test; error bars show standard deviation; P values are as shown; n = 10.
(TIF)

Figure S2 **Knockdown of SOD2 reduces the level of E-cadherin.** Histogram showing the relative level of E-cadherin expression was significantly greater in control (+) lymph glands than in those with knockdown of SOD2 during the early-third instar. Student's t-test; error bars show standard deviation; P values are as shown; n = 14.
(TIF)

Figure S3 **Loss of SOD2 reduces the number of Odd-expressing prohemocytes in late-third instar lymph glands.** Odd-expressing prohemocytes in (**A**) control and (**B**) *Sod2/Sod2* hypomorphic *(Sod2)* lymph glands from late-third instar larvae. White dotted lines delineate the entire lymph gland; yellow dotted lines delineate the prohemocyte pool. (**C**) Histogram showing the percentage of Odd-expressing prohemocytes was significantly reduced in *Sod2* lymph glands compared to controls (+). Student's t-test; error bars show standard deviation; P values are as shown; n = 14.
(TIF)

Figure S4 **Paraquat treatment increases ROS levels and reduces E-cadherin expression in the lymph gland.** (**A,B**) ROS levels were measured using the superoxide specific dye, dihydroethdium (DHE). ROS levels increased in the lymph glands of (**B**) paraquat-treated (10 mM) compared to (**A**) untreated (0 mM) controls. (**C,D**) E-cadherin expression in the lymph gland was assessed in paraquat treated larvae. (**D**) Paraquat treatment (10 mM) reduces the level of E-cadherin expression compared to (**C**) untreated (0 mM) controls. White dotted lines delineate the entire lymph gland; yellow dotted lines delineate the prohemocyte pool.
(TIF)

Figure S5 **Knockdown of SOD2 results in increased levels of Srp expression.** Histogram showing the relative levels of Srp expression in control (+) lymph glands and those with SOD2 knocked down (*Sod2^RNAi^*) during the early-third instar. Student's t-test; error bars show standard deviation; P values are as shown; n = 15.
(TIF)
