# Supplementary figures and images for: Antioxidants Maintain E-Cadherin Levels to Limit Drosophila Prohemocyte Differentiation
Source: PLoS One. 2014 Sep 16;9(9):e107768. doi: 10.1371/journal.pone.0107768 (PMC4167200; doi:10.1371/journal.pone.0107768)

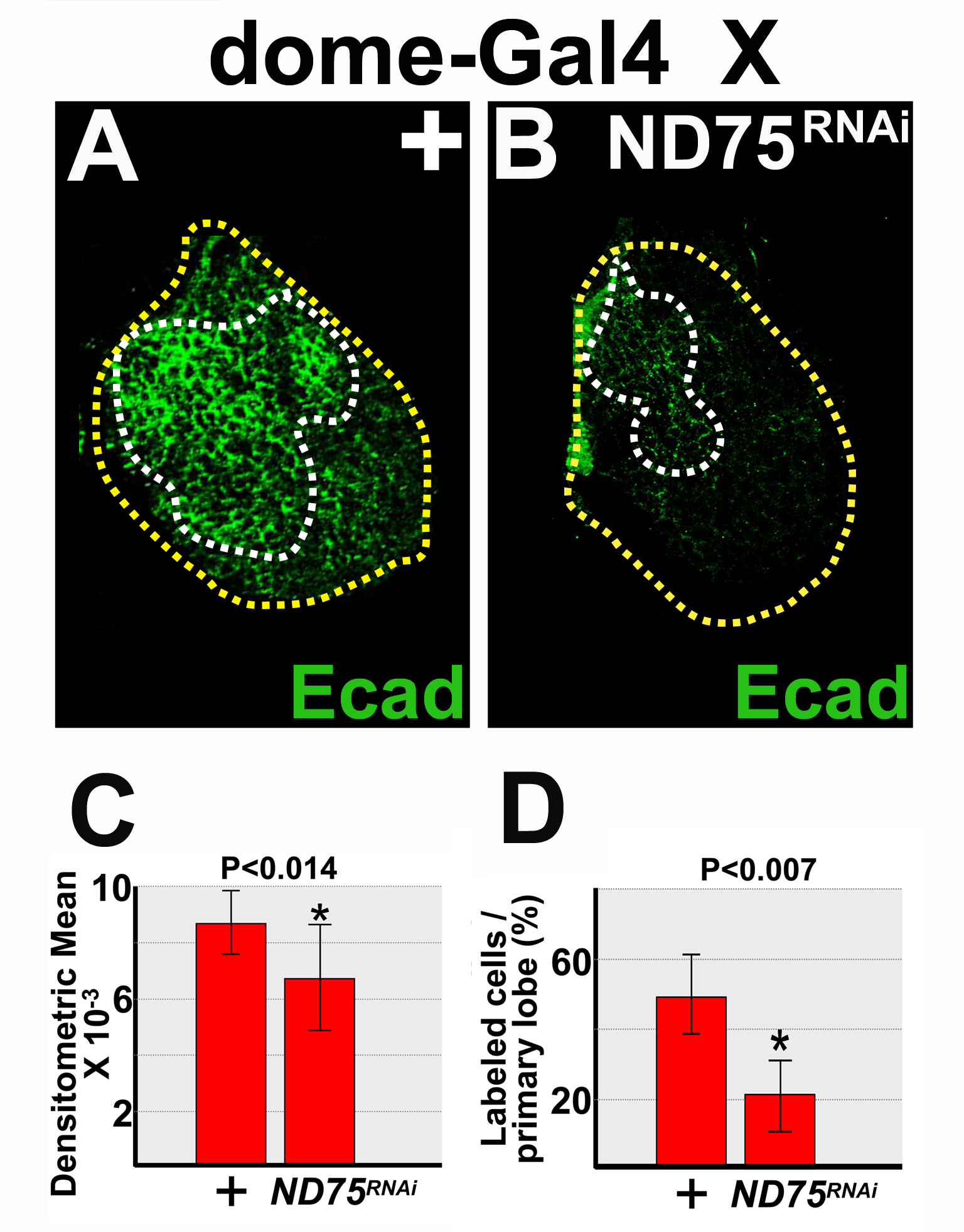

Supplement: Figure S1 — Knockdown of ND75 in prohemocytes reduces E-cadherin expression. E-cadherin expression is greater in (A) control than in (B) ND75 knockdown (ND75RNAi) lymph glands. dome-Gal4 females were crossed to UAS-ND75RNAi or wild-type (+) males. Yellow dotted lines delineate the entire lymph gland; white dotted lines delineate the prohemocyte pool. (C) Histogram showing the relative level of E-cadherin expression was significantly greater in control (+) lymph glands than in those with knockdown of ND75. (D) Histogram showing the percentage of E-cadherin-expressing prohemocytes was significantly reduced in ND75RNAi lymph glands compared to controls (+). Student's t-test; error bars show standard deviation; P values are as shown; n = 10. (TIF) [file pone.0107768.s001.tif]

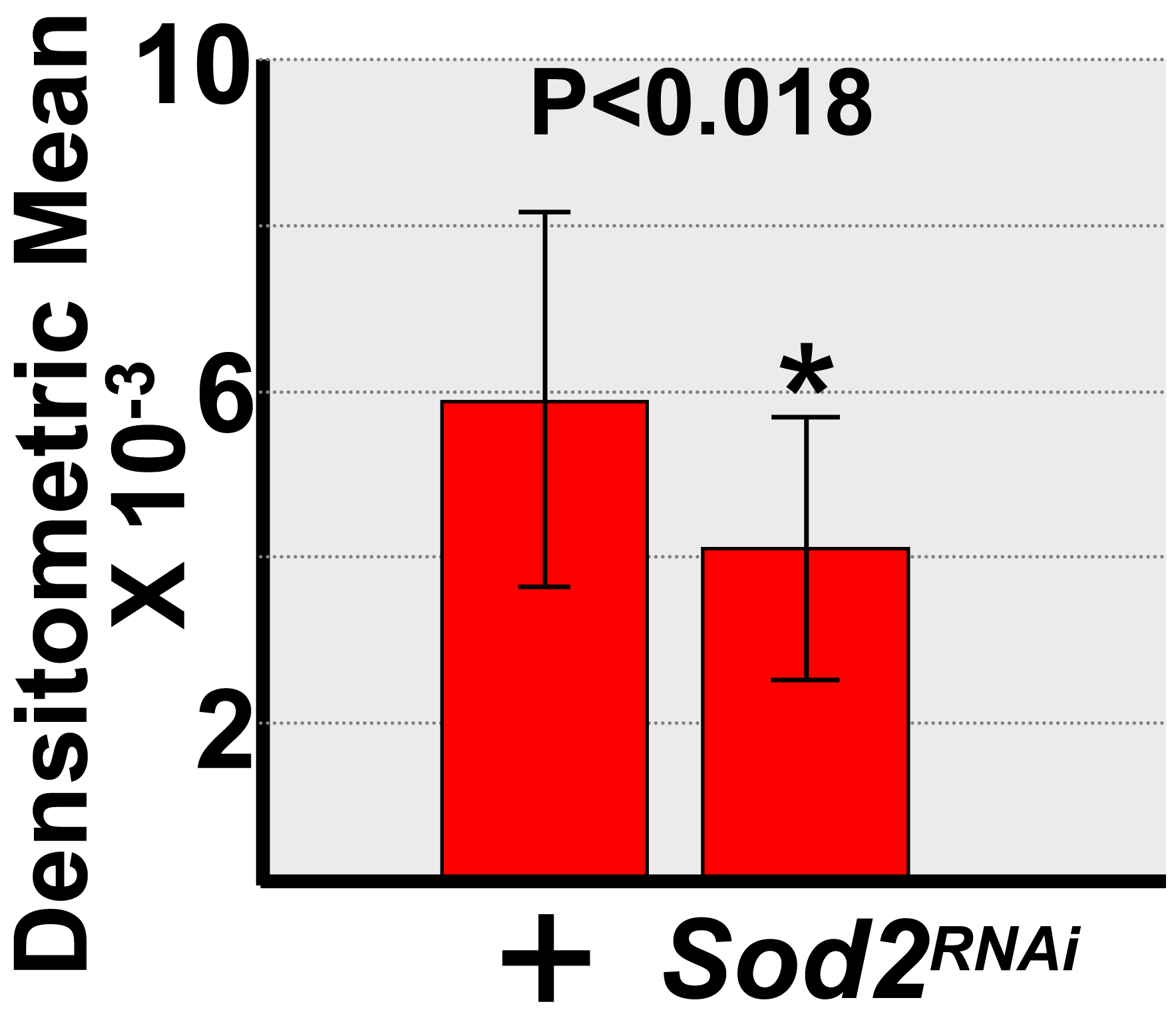

Supplement: Figure S2 — Knockdown of SOD2 reduces the level of E-cadherin. Histogram showing the relative level of E-cadherin expression was significantly greater in control (+) lymph glands than in those with knockdown of SOD2 during the early-third instar. Student's t-test; error bars show standard deviation; P values are as shown; n = 14. (TIF) [file pone.0107768.s002.tif]

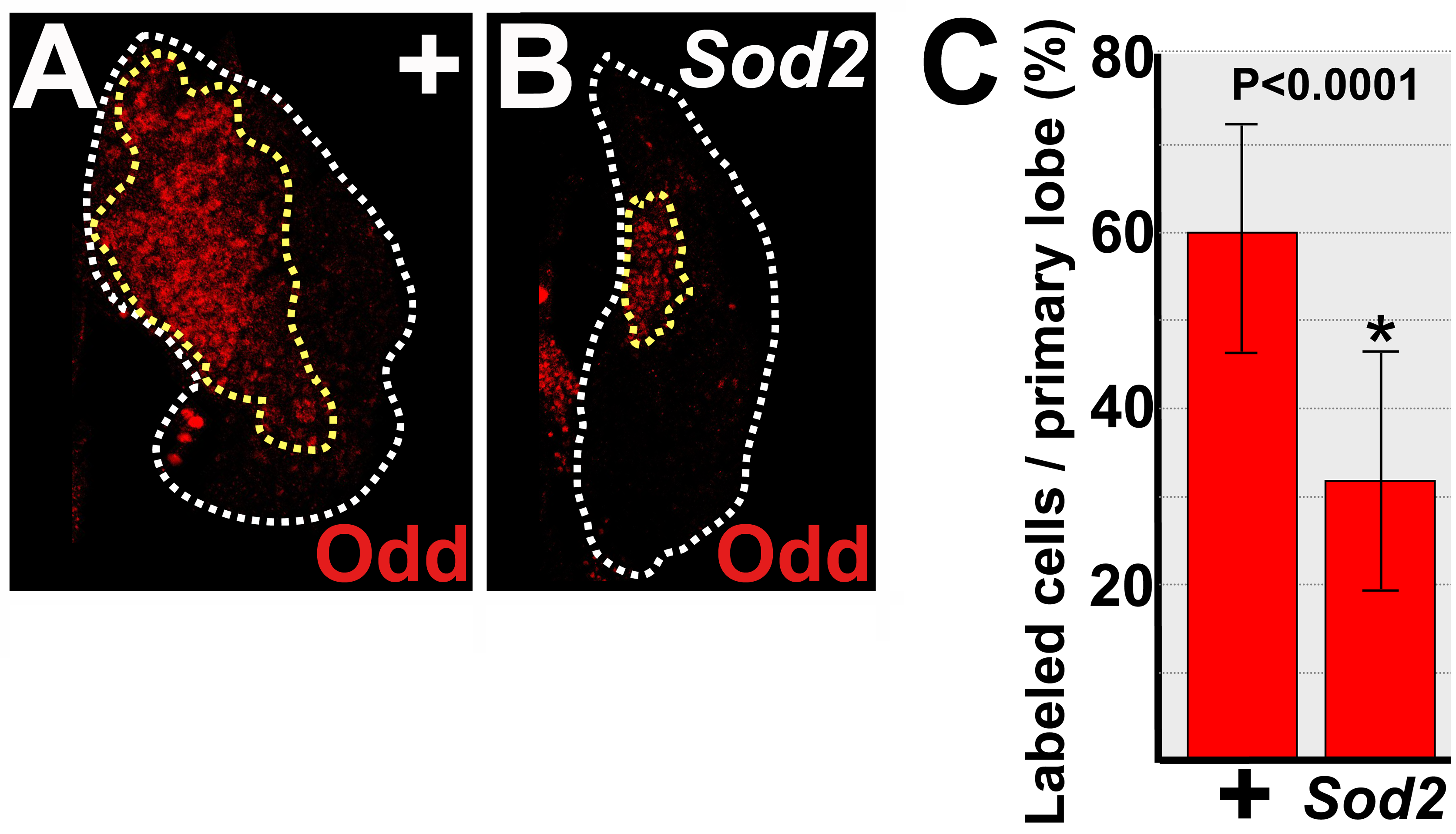

Supplement: Figure S3 — Loss of SOD2 reduces the number of Odd-expressing prohemocytes in late-third instar lymph glands. Odd-expressing prohemocytes in (A) control and (B) Sod2/Sod2 hypomorphic (Sod2) lymph glands from late-third instar larvae. White dotted lines delineate the entire lymph gland; yellow dotted lines delineate the prohemocyte pool. (C) Histogram showing the percentage of Odd-expressing prohemocytes was significantly reduced in Sod2 lymph glands compared to controls (+). Student's t-test; error bars show standard deviation; P values are as shown; n = 14. (TIF) [file pone.0107768.s003.tif]

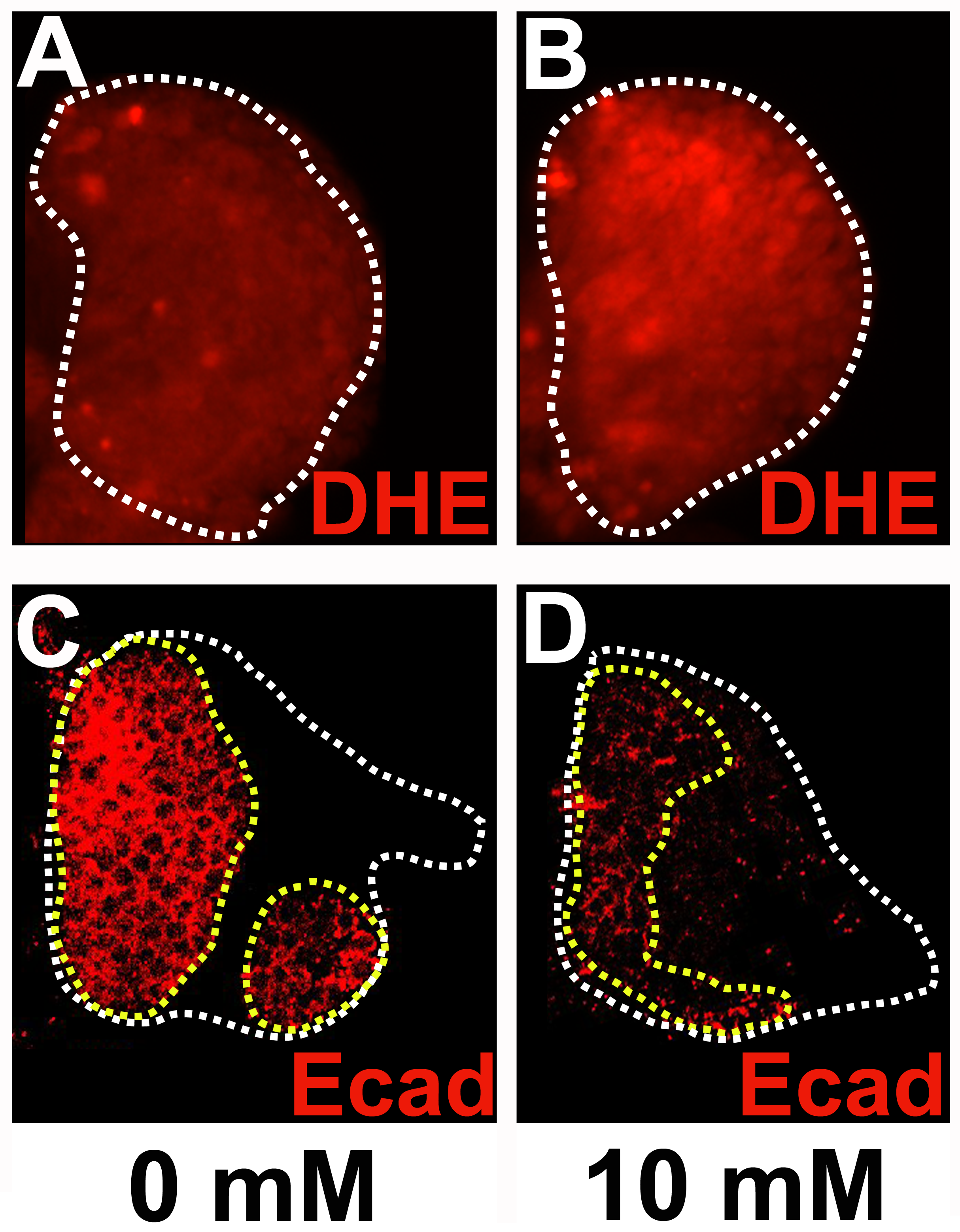

Supplement: Figure S4 — Paraquat treatment increases ROS levels and reduces E-cadherin expression in the lymph gland. (A,B) ROS levels were measured using the superoxide specific dye, dihydroethdium (DHE). ROS levels increased in the lymph glands of (B) paraquat-treated (10 mM) compared to (A) untreated (0 mM) controls. (C,D) E-cadherin expression in the lymph gland was assessed in paraquat treated larvae. (D) Paraquat treatment (10 mM) reduces the level of E-cadherin expression compared to (C) untreated (0 mM) controls. White dotted lines delineate the entire lymph gland; yellow dotted lines delineate the prohemocyte pool. (TIF) [file pone.0107768.s004.tif]

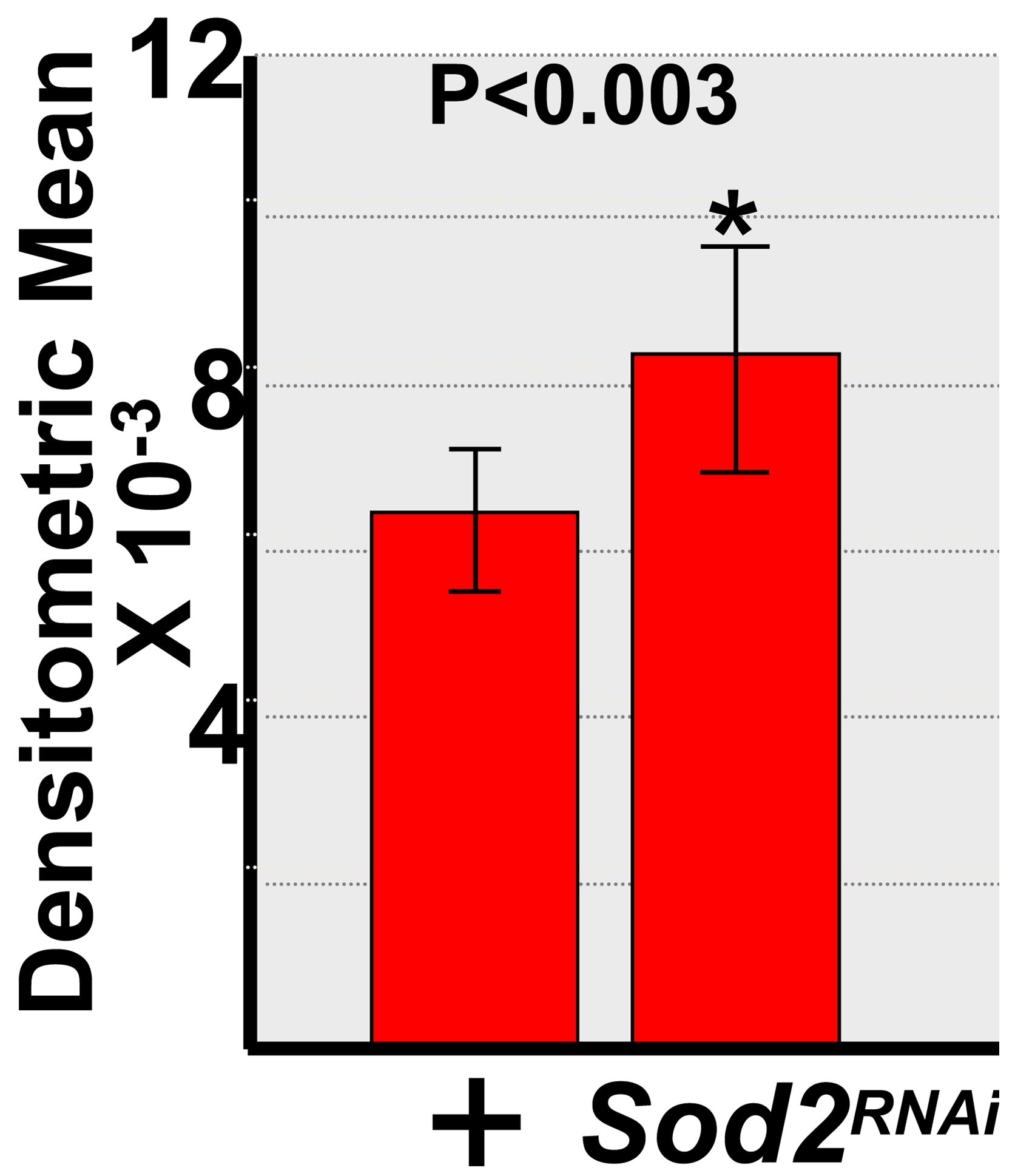

Supplement: Figure S5 — Knockdown of SOD2 results in increased levels of Srp expression. Histogram showing the relative levels of Srp expression in control (+) lymph glands and those with SOD2 knocked down (Sod2RNAi) during the early-third instar. Student's t-test; error bars show standard deviation; P values are as shown; n = 15. (TIF) [file pone.0107768.s005.tif]
